# Intranasal Insulin Reduces White Matter Hyperintensity Progression in Association with Improvements in Cognition and CSF Biomarker Profiles in Mild Cognitive Impairment and Alzheimer's Disease

**DOI:** 10.14283/jpad.2021.14

**Published:** 2021-04-07

**Authors:** D. Kellar, S.N. Lockhart, P. Aisen, R. Raman, R.A. Rissman, J. Brewer, Suzanne Craft

**Affiliations:** 1 Department of Internal Medicine-Geriatrics, Wake Forest School of Medicine, One Medical Center Boulevard, 27157, Winston-Salem, NC, USA; 2 Alzheimer's Therapeutic Research Institute, University of Southern California, San Diego, USA; 3 Department of Neurosciences, University of California, La Jolla, San Diego, USA

**Keywords:** Alzheimer's disease, clinical trial, intranasal insulin, white matter, CSF

## Abstract

**Background:**

Intranasally administered insulin has shown promise in both rodent and human studies in Alzheimer's disease; however, both effects and mechanisms require elucidation.

**Objective:**

We assessed the effects of intranasally administered insulin on white matter health and its association with cognition and cerebral spinal fluid biomarker profiles in adults with mild cognitive impairment or Alzheimer's disease in secondary analyses from a prior phase 2 clinical trial (NCT01767909).

**Design:**

A randomized (1:1) double-blind clinical trial.

**Setting:**

Twelve sites across the United States.

**Participants:**

Adults with mild cognitive impairment or Alzheimer's disease.

**Intervention:**

Participants received either twice daily placebo or insulin (20 IU Humulin R U-100 b.i.d.) intranasally for 12 months. Seventy-eight participants were screened, of whom 49 (32 men) were enrolled.

**Measurements:**

Changes from baseline in global and regional white matter hyperintensity volume and gray matter volume were analyzed and related to changes in cerebral spinal fluid biomarkers, Alzheimer's Disease Assessment Scale-Cognition, Clinical Disease Rating-Sum of Boxes, Alzheimer's Disease Cooperative Study-Activities of Daily Living Scale, and a memory composite.

**Results:**

The insulin-treated group demonstrated significantly reduced changes in white matter hyperintensity volume in deep and frontal regions after 12 months, with a similar trend for global volume. White matter hyperintensity volume progression correlated with worsened Alzheimer's disease cerebral spinal fluid biomarker profile and cognitive function; however, patterns of correlations differed by treatment group.

**Conclusion:**

Intranasal insulin treatment for 12 months reduced white matter hyperintensity volume progression and supports insulin's potential as a therapeutic option for Alzheimer's disease.

## Introduction

**A**lzheimer's disease (AD) is the leading cause of dementia and, as there are currently no disease modifying treatments, its prevalence is expected to increase in response to an aging population (1). AD is characterized by aggregation of amyloid beta (Aβ) plaques and tau neurofibrillary tangles (NFT). Clinical trials have attempted to reduce accumulation of these proteins in the brain and prevent further cognitive decline; however, although some amyloid antibody trials have successfully reduced plaque load, none have been successful to date in halting the progression of AD symptoms (2, 3). Positron Emission Tomography (PET) measures AD pathology by quantifying the load of Aβ and NFT in the brain, and a number of studies have demonstrated relationships between PET measures and concentrations of these proteins in cerebral spinal fluid (CSF) (4, 5). CSF Aβ42 decreases as amyloid accumulation in brain increases, suggesting it is being sequestered in the brain parenchyma, while hyperphosphorylated tau (p-tau) in CSF increases with increased propagation of NFT (6). Utilizing ratios of Aβ42 to Aβ40, p-tau, and total tau (t-tau) has been found to further improve specificity and sensitivity to identify AD dementia (7, 8). There remains a clear need for a pharmacological intervention to prevent or slow AD progression.

As AD progresses, gray matter volume is reduced. While this is also true of non-pathological aging, adults with AD exhibit a far greater reduction in overall gray matter associated with a predictable pattern (9). The hippocampus and entorhinal cortex are perhaps the most easily detected regions affected by AD (10); however, an AD-defined meta region including the hippocampus and other regions such as entorhinal, inferior temporal, middle temporal, inferior parietal, fusiform, and precuneus has recently been utilized in order to increase sensitivity (11). Cortical thickness has also been postulated to be a more reliable marker of AD progression as it is less affected by total intracranial volume which can vary greatly between patients (11). While progression of the disease and corresponding gray matter reduction have been well established, its utility as a clinical trial endpoint needs to be validated. Interventions that have successfully removed amyloid or even allayed cognitive decline have also been associated with reduced gray matter volume (12, 13). These findings illustrate a complex relationship between brain health and gray matter volumetrics which is not fully understood.

White matter integrity can be reflected by the presence of white matter hyperintensities (WMH) detected with magnetic resonance imaging (MRI); however, the relationship of WMHs and AD progression has not been characterized in detail. WMHs are detected using fluid-attenuated inversion recovery (FLAIR) MRI, are presumed to indicate cerebrovascular pathology (14), and are associated with gliosis, demyelination, axonal loss, and arteriosclerosis (15). It is postulated that WMHs reflect a number of factors including hypoxia, amyloid angiopathy, blood brain barrier damage, degeneration, hypoperfusion, and inflammation (15). WMH volume (WMHV) increases with age and some studies have found WMHV to be independent of Aβ burden, leading to the proposal that WMHs should be considered a co-pathology that do not directly contribute to AD (16, 17). Other studies have found correlations between WMHs and cortical tau load in AD (18). It has been proposed that cerebrovascular pathology represented by WMHs precedes and therefore could initiate Aβ aggregation (19, 20, 21). Conversely, other investigators claim that Aβ induces vascular damage through neuroinflammation, formation of reactive oxygen species, and oxidative stress (22, 23). It is possible that vascular and AD-specific pathology form a vicious cycle giving rise to these differing viewpoints, or that the precise time course of the association differs for subgroups of patients with AD.

Identification of the nature of the association of WMHs and AD is hindered by lack of a commonly-accepted standardized approach to measurement of WMHs (24). Methods evaluating WMHs range from semi-quantitative visual reads using one of 3 established rating scales (Manolio, Fazekas and Schmidt, Scheltens) to fully automated lesion segmentation (25, 26). Representation of the data is also not consistent, with some studies reporting global ratings or volumes and others focusing on spatial patterns (27, 28). There are also many ways to spatially segment WMHs such as periventricular versus deep (29); however, deep can also be further split in to juxtacortical and non-juxtacortical[30]. Studies in which WMHs are segmented in classical lobular fashion have reported that AD is associated with temporal WMHs (31), or with global and parietal/occipital volumes (32). Volumes can also be displayed as raw volumes (33), log transformed values (33), or percent change ratios (34, 35). As the field progresses automated techniques generating quantitative spatially accurate information may prove the best way to track WMH progression in AD. It is clear that WMHs are associated with poor cognitive outcomes and preventing progression is a clinically relevant marker.

A promising area of research in the treatment and prevention of AD focuses on metabolism, inflammation and, in particular, the role of insulin in the central nervous system. With respect to metabolism, although insulin does not appear to impact global transport of glucose into the brain, it has been shown to increase glucose uptake via the glucose transporter GLUT4 in selected regions such as the hippocampus (36). Further, insulin increases glycogen storage in astrocytes, thereby providing an alternate energy source during glucose deprivation or intense neuronal activity (37). Insulin has long been implicated in AD and several reviews have highlighted both its importance and therapeutic potential (38, 39, 40). In short, insulin has been demonstrated to modulate both Aβ and pathological tau formation, and improves neuronal health, dendritic spine proliferation, and white matter integrity. Insulin can be administered intranasally where it is detectible in perivascular spaces with PET imaging (41) and in the CSF in less than 30 minutes (42). A promising pilot trial documented improvements in delayed memory recall, preserved Alzheimer's Disease Assessment Scale (ADAS-Cog) scores, and functional abilities assessed by the Alzheimer's Disease Cooperative Study-Activities of Daily Living Scale (ADCS-ADL) after 4 months of treatment with intranasal insulin compared to placebo (43). A recent large 18-month phase II clinical trial of INI treatment in AD and MCI patients found differing patterns of results depending on the device used to administer the insulin (13). For the device used by the primary intent-to-treat cohort, no significant differences in rates of decline measured by the ADAS-Cog13, Clinical Disease Rating-Sum of Boxes (CDR-SOB), ADCS-ADL, or CSF Aβ and tau were observed between placebo and insulin groups. In a secondary cohort, a different device showed better performance on ADAS-Cog13 in the insulin-treated group compared to placebo at 6 months with a similar trend at 12 months. In open-label analyses, the early-start secondary device cohort treated with insulin performed better on the ADAS-Cog13 and ADL-MCI at 18 months than the delayed start secondary group. The insulin-treated group using this device also demonstrated an improvement in CSF Aβ42/Aβ40 and Aβ42/t-tau ratios at 12 months. This study highlights the need for additional investigation to definitively determine the potential for intranasal insulin as a therapeutic for AD.

In the present study, we assessed the effects of INI on white matter health in the secondary cohort of participants using the device associated with improved cognition and AD biomarker profiles. There are several mechanisms through which insulin could act directly to improve white matter health and prevent WMH progression (44). Reduced insulin levels or activity impair oligodendrocyte myelin survival and maintenance, and increase ceramides and decreases sulfatides, leading to oxidative stress, inflammation, and lipid peroxidation. These factors all contribute to myelin damage and subsequent WMHs. Insulin resistance impairs vascular responsiveness, causing luminal narrowing and fibrosis, which cause decreased blood flow and blood brain barrier damage. These effects lead to ischemia and inflammation and promote the formation of WMHs. As mentioned previously, insulin reduces Aβ and p-tau levels in the brain, both of which can cause inflammation, neuronal and glial damage, and vascular impairment (45). These distinct pathways could all culminate in the formation and progression of WMHs, thus poising insulin at a convergence point in several potential cascades, and raising the possibility that providing insulin to the brain to overcome deficient insulin availability or activity may have therapeutic benefit in AD.

Based on this evidence, we examined the effect of 12 months of INI treatment vs. placebo on change in WMHs. We also examined the relationships among changes in WMHs, cognition, and AD CSF biomarkers.

## Methods

The study was overseen by the Alzheimer's Therapeutic Research Institute (P. Aisen, Director) together with the Principal Investigator (S. Craft). Eligibility and recruitment for this study have been described previously (13). Two devices were used in the parent study; however, only one device demonstrated cognitive benefits or changes in AD CSF biomarkers across the 18-month long study. For this reason, we evaluated only the group using the device that showed potential beneficial effects. Briefly, participants with AD (n=31) or amnestic MCI (n=18) were recruited from 12 sites. Participants received baseline testing including CDR, MMSE, ADAS-Cog13, a lumbar puncture, and an MRI, then were randomized on a 1:1 basis to receive either 20 IU intranasal insulin (n=24) or placebo (n=25) twice daily for 12 months. After 12 months the cognitive battery was readministered, a lumbar puncture was performed, and another MRI was obtained. There were a total of 40 participants (insulin n=20; placebo n=20) with MRI data that passed quality control measures at baseline and month 12.

T1 and Fluid Attenuated Inversion Recovery images were collected with 1.5 or 3T MRI. T1 weighed images were processed using FreeSurfer 6.0.0 to produce participant specific gray matter volume, thickness, and area. FLAIR images were segmented by the lesion growth algorithm[46] as implemented in the LST toolbox version 3.0.0 (www.statisticalmodelling.de/lst.html) for SPM. The algorithm first segments the T1 images into the three main tissue classes (CSF, GM and WM). This information is then combined with the coregistered FLAIR intensities in order to calculate lesion belief maps. By thresholding these maps with a pre-chosen initial threshold (κ= 0.3) an initial binary lesion map is obtained which is subsequently grown along voxels that appear hyperintense in the FLAIR image. The result is a lesion probability map. The lesion probability maps were then warped to MNI space and lobular volume was extracted using Mayo Clinic Adult Lifespan Template (47). A temporal-parietal volume meta-ROI was created to examine volume and was defined as bilateral entorhinal, inferior temporal, middle temporal, inferior parietal, fusiform, and precuneus (11). Cortical thickness was similarly defined by Jack (48) as the surface area weighted thickness of the entorhinal, inferior temporal, middle temporal, and fusiform.

CSF was collected in the morning after an overnight fast and was immediately placed on dry ice and was shipped overnight to the central biomarker laboratory. AD biomarkers Aβ42, Aβ40, total tau, and tau phosphorylated at threonine 181 were quantified with the Meso Scale Discovery platform (Meso Scale Diagnostics). Blood was collected for APOE genotyping using established protocols.

Cross sectional analysis was performed to assess group differences at baseline in age, cognitive status, sex, baseline surface weighted cortical thickness, AD signature region volume, total WMHV, and regional WMHV at baseline using general linear models or chi squared tests when appropriate. Change variables for gray matter and WMHV were defined as percentage change from baseline as previously described (35). General linear modeling was performed in SAS v 9.4 with covariates age, ApoE4 status, study site, and sex included in all initial models. Baseline volumes were also included for GMV and WMHV models, and total intracranial volume was included in models analyzing data in native space. Non-contributing covariates (p>0.15) were dropped from the model. No adjustments were made for multiple comparisons; rather, results are reported as mean estimates and corresponding 95% confidence intervals. In exploratory analyses, we examined whether individual treatment groups showed reliable change in WMHV over time with within-group LSMEANS t-tests. Change variables for WMHV, cognitive scores, and CSF values were subjected to Pearson's r correlations to determine inter-relationships.

## Results

### Participants

**Figure 1:**
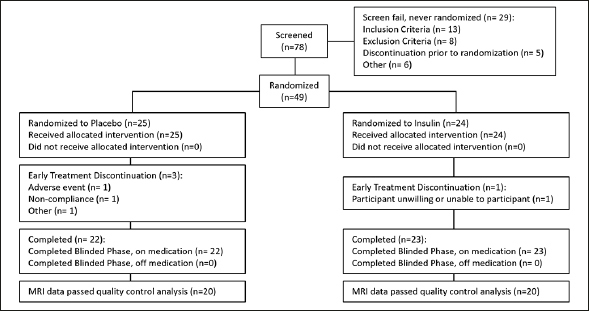
CONSORT diagram

For the parent study secondary cohort that utilized the device associated with cognitive benefit, 78 participants were screened, of whom 49 (32 men [65.3%]) were enrolled. Twenty-four were randomized to the insulin arm and 25 were randomized to the placebo arm (Figure 1). Of those 49, 40 participants (insulin n=20, placebo n=20) had usable MRI data at both time points and were analyzed for this study. There were no demographic or other notable clinical differences between participants with usable and unusable data. There were also no differences in demographic characteristics between arms at baseline (Table 1).

**Table 1:** Descriptive characteristics of cohort

	Placebo n = 20	Insulin n = 20	Combined n = 40
Gender			
Male	14 (70%)	11 (55%)	25 (62%)
Female	6 (30%)	9 (45%)	15 (38%)
Diagnosis			
AD	15 (75%)	11 (55%)	26 (65%)
MCI	5 (25%)	9 (45%)	14 (35%)
Racial Categories			
Black or African American	0 (0%)	1 (5%)	1 (2%)
White	20 (100%)	19 (95%)	39 (98%)
APOE-ε4 Carriage			
No	4 (20%)	5 (25%)	9 (23%)
Yes	16 (80%)	15 (75%)	31 (77%)
Age, years (SD)	70.88 (5.69)	71.69 (8.25)	71.28 (7.01)
Education, years (SD)	17.2 (2.48)	16.05 (2.87)	16.62 (2.71)
Baseline ADAS-cog	24.75 (8.75)	24.15 (7.36)	24.45 (7.99)
Baseline ADL	39.65 (7.35)	42.65 (7.23)	41.15 (7.35)
Baseline CDR-SOB	3.15 (1.65)	2.7 (1.36)	2.92 (1.51)

### MRI Results

**Figure 2:**
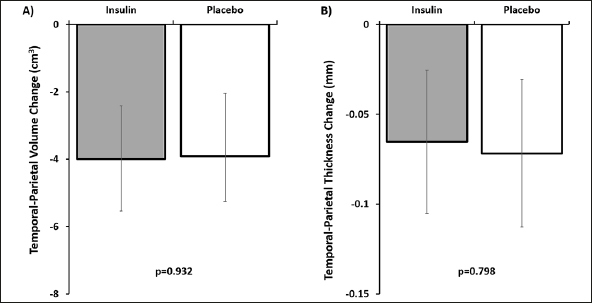
Changes in gray matter A) volume and B) surface weighted thickness in the temporal-parietal meta-ROI There were no significant differences between treatment group and placebo. Error bars represent 95% confidence intervals.

The temporal-parietal meta-ROI decreased in volume over time as did the surface weighted cortical thickness (both ps<.001, Figure 2). There was no interaction between treatment arm and rate of decline of gray matter or cortical thickness (Supplementary Table 1).

**Figure 3:**
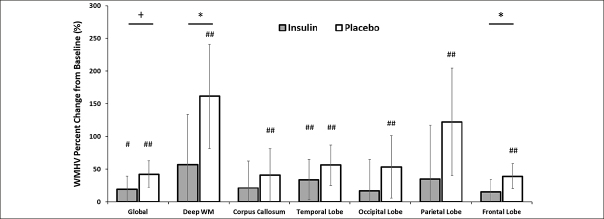
White Matter Hyperintensity Volume (WMHV) as percent change from baseline both globally and regionally split by MCALT (excluding cerebellum and midbrain regions) There were significant differences between the degree of change for insulin and placebo groups in the deep white matter and frontal regions with a similar trend for global change (+ p<0.10, * p<0.05). The placebo group showed significantly increased change from baseline WMHV in all regions, whereas the insulin group showed significant change only in temporal lobe with a trend for global change (# <0.10, ## p<0.05). Error bars represent 95% confidence intervals.

An interaction between treatment arm and global WMHV was observed such that the insulin-treated arm tended to have less global WMH volume increase over the 12 month intervention compared to the placebo group (insulin lsmeans [95% CI]=18.98 [−1.38,39.33] and placebo 42.21 [21.70,62.72], p=0.064, Figure 3). Given this trend, exploratory analyses were conducted for comparisons of individual ROIs between treatment arms. Insulin significantly reduced WMHV change over the 12 month intervention in both the frontal lobe and deep white matter compared to placebo (frontal insulin lsmeans [95% CI]=15.14 [−3.84,34.12] and placebo=39.18 [20.05,58.30], p=0.042; deep WM insulin lsmeans [95% CI]=56.94 [−20.20,134.08] and placebo=161.37 [81.68, 241.05], p=0.042, Figure 3). Change in WMHV was less in the insulin arm than the placebo arm for all other regions, although these comparisons did not reach statistical significance.

When we examined whether individual treatment groups showed reliable change in WMHV over time with within-group LSMEANS t-tests, the placebo group showed significantly increased WMHVs across all regions (all ps<0.05, Figure 3; raw means for baseline and month 12 for all regions are presented in Supplementary Table 2), whereas WMHV was unchanged following insulin treatment in the deep white matter, corpus callosum, occipital, parietal, and frontal regions (all p>0.1, Figure 3). Temporal WMHV increased slightly over the 12 month intervention with insulin treatment with a similar trend in global WMHV (temporal p=0.033; global p=0.066), although to a lesser degree than with placebo.

### Correlation between MRI and Cognitive Outcomes

**Figure 4:**
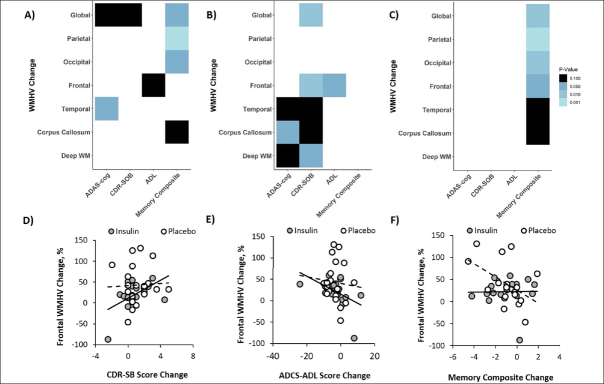
Changes in global and regional White Matter Hyperintensity Volume (WMHV) correlate with changes in ADAS-cog, CDR-SB, ADCS-ADL, and a memory composite Analyses were performed for both insulin and placebo groups combined (A) and for the insulin treatment arm (B) and placebo (C) groups independently. Light colors represent correlations with lower p values (ps range from <0.001 to 0.10 from light to dark). Exemplar scatterplots are shown that demonstrate relationships between change in frontal WMHVs (which differed between insulin and placebo groups) and change in (D) CDR-SB, (E) ADCS-ADL, and (F) memory composite scores.

For the combined cohort including both insulin and placebo groups, increased global WMHV correlated with lowered memory composite scores (r=−0.38, p=0.024, Figure 4A) and similar trends were observed for the ADAS-Cog13 and CDR-SOB (r=0.297, p=0.062; r=0.278, p=0.081, figure 4A). Regional analysis revealed a significant correlation between the memory composite score and parietal and occipital WMHVs and a trend correlation for the corpus callosum (r=−0.536, p=0.001; r=−0.405, p=0.015; r=0.31, p=0.069, Figure 4A). Increased temporal WMHV was associated with worsened (higher) scores for the ADAS-Cog13 (r=0.313, p=0.049, Figure 4A). A similar trend correlation was observed between frontal WMHV and ADCS-ADL scores (r=−0.267, p=0.095, Figure 4A).

When analyzed by treatment group, the insulin group showed a significant correlation between change in ADAS-Cog13 scores and WMHVs change in the corpus callosum, and trending correlations for the deep white matter and temporal regions (r=0.459, p=0.041; r=0.409, p=0.073; r=0.368, p=0.092, Figure 4B). Increased (worsened) CDR-SOB scores correlated significantly with increased frontal and global WMHV with trends noted for deep white matter, temporal, and the corpus callosum regions (r=0.535, p=0.014; r=0.537, p=0.014; r=0.439, p=0.052; r=0.426, p=0.059; r=0.409, p=0.061, Figure 4B). Smaller increases in frontal WMHV also correlated with smaller increases in ADCS-ADL scores (r=−0.475, p=0.034). In the placebo group, declines in memory composite scores only correlated with increased WMHV in global, parietal, and occipital regions with trends for frontal, corpus callosum, and temporal regions (r=−0.639, p=0.005; r=−0.773, p=0.001; r=−0.585, p=0.013; r=−0.48, p=0.05; r=0.435, p=0.08; r=−0.425, p=0.088, Figure 4C).

### Correlation between MRI and CSF outcomes

**Figure 5:**
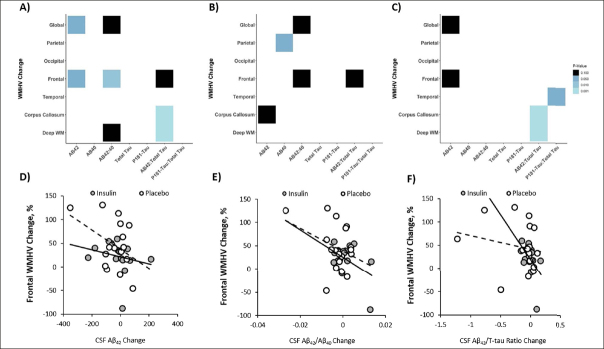
Changes in global and regional White Matter Hyperintensity Volume (WMHV) correlate with changes in CSF AD biomarkers Analyses were performed for both insulin and placebo groups combined (A) and for the insulin treatment arm (B) and placebo (C) groups independently. Light colors represent correlations with lower p values (ps range from <0.001 to 0.10 from light to dark). Exemplar scatterplots are shown that demonstrate relationships between change in frontal WMHVs (which differed between insulin and placebo groups) and change in CSF (D) Aβ42, (E) Aβ42/Aβ40 ratio, and (F) Aβ42/T-tau ratio.

For the combined cohort including both insulin and placebo groups, global WMHV increase correlated with a decrease in CSF Aβ42 and with a similar trend for the Aβ42/Aβ40 ratio (r=−0.375, p=0.028; r=−0.328, p=0.058, Figure 5A). Frontal WMHV increases also correlated with decreases in both Aβ42 and Aβ42/Aβ40 ratio (r=−0.355, p=0.039; r=−0.44, p=0.009, Figure 5A). Increases in both corpus callosum and deep white matter WMHV correlated with decreases in Aβ42/tau ratio (r=−0.397, p=0.001; r=−0.734, p=0.001, figure 5A).

When analyzed by treatment group, there was a significant correlation between increased parietal WMHV and decreased Aβ40 in the insulin group (r=−0.525, p=0.036, figure 5B). There were trending relationships between increased global WMHV and decreased Aβ42/ Aβ40 ratio, increased frontal WMHV and decreased Aβ42/Aβ40 ratio and Aβ42/t-tau ratio changes, and increased corpus callosum and decreased Aβ42 levels (r=−0.443, p=0.085; r=−0.467, p=0.067; r=−0.477, p=0.061; r=−0.446, p=0.083, figure 5B). In the placebo group, increases in both corpus callosum and deep white matter WMHV correlated with decreases in Aβ42/t-tau ratio (r=−0.733, p=0.001; r=−0.724, p=0.001, figure 5C). Temporal WHV change positively correlated with ptau-181/t-tau ratio (r=0.481, p=0.043, figure 5C). There was a trend for both global and frontal WMHV change to negatively correlate with Aβ42 change (r=−0.431, p=0.073; r=−0.408, p=0.092, figure 5C).

## Discussion

The present study found that increased WMHV correlated with greater declines in cognition and worsening of CSF AD biomarker profiles, and that INI treatment for 12 months reduced WMHV progression in key brain regions. White matter hyperintensities represent cerebral small vessel disease and white matter damage resulting from degraded myelin, and have been shown to increase with aging. Some studies have suggested that regional, and not global WMHV may best predict, or correlate, with AD progression (49). While there are numerous ways to segregate WMHs the most promising research has suggested a division between frontal and posterior regions in differentiating normal versus pathological aging, with lobular divisions further increasing the regional specificity (32, 50). We observed that INI treatment for 12 months slowed the progression of WMHs globally and in deep WM and frontal regions compared to placebo assignment. This finding supports previous studies linking frontal WMHs and pathological aging and suggests that INI may reduce AD-related WMH progression in these key areas. Exploratory analysis also demonstrated that for all other regions, with the exception of the temporal lobe, progression of WMHs were stabilized following insulin treatment. These findings may be considered clinically significant as growing research has shown that WMHVs correlate with numerous pathological outcomes such as ependymal loss, cerebral ischemia, and demyelination (51). Insulin could be acting through a number of pathways to preserve white matter health (44). Insulin resistance impairs oligodendrocyte myelin maintenance and survival, while also leading to reduced vascular smooth muscle responsiveness. Increasing insulin availability could ameliorate these deficiencies thereby reducing WMH progression.

Previous research has shown that higher baseline WMHV predicts worse cognitive outcomes measured by the ADAS-Cog (52) and CDR (53). We demonstrated in the combined cohort that over 12 months, longitudinal increases in WMHV correlate with decreases in cognition measured by the ADAS-Cog, CDR-SOB, ADCS-ADL, and a memory composite. We also found that correlations differed depending on treatment group. The placebo group showed correlations only between WMHV and memory composite scores, while the insulin treated group showed associations between WMHs and both the ADAS-Cog13 and CDR-SOB, as well as the ADCS-ADL. Of note, in the parent study, insulin treatment was shown to benefit performance on the ADAS-Cog13, CDR, and ADCS-ADL for the secondary cohort (13). The present findings raise the possibility that insulin's effects on these measures are mediated in part by factors associated with WMHV. These findings suggest that preventing or even delaying progression of white matter damage could prevent global cognitive and functional decline.

Increased CSF Aβ42 levels and decreased hyperphosphorylated tau concentrations have been proposed as markers demonstrating improvement in AD pathology (54, 55). Studies have shown that greater Aβ load, indicated by lower CSF Aβ42 concentration and increased Aβ PET, correlates with greater WMH burden (56, 57, 58, 59); however, the findings regarding p-tau and total tau are less consistent, with some studies reporting correlations (18, 60) while other do not (59, 61, 62). In the parent study, insulin treatment was associated with improved (increased) Aβ42/Aβ 40 and Aβ42/tau ratios (13) for the secondary cohort treated with the device which was associated with cognitive benefit. The present study adds to these findings by demonstrating that WMHV changes inversely correlate with changes in Aβ40, Aβ42, Aβ42/Aβ40 ratio, and Aβ42/t-tau ratio; however, these associations differ by region and CSF metric. The relationship between WMHV and tau and Aβ may change throughout the course of the disease as Aβ pathology is stabilized and tau pathology increases; however, this hypothesis is still in contention (59). Insulin affects the regulation of both Aβ and hyperphosphorylated tau (63). Both Aβ40 (64) and Aβ42 (65) cause inflammation via production of reactive oxygen species. Aβ42 aggregates and starts seeding points which grow to plaques leading to death in several cell types including oligodendrocytes (66, 67), while Aβ40 is more prone to form deposits on vessels walls where it damages pericytes (68, 69). Hyperphosphorylated tau also leads to neurovascular dysfunction resulting in reduced energy supply due to aberrant vessel dilation (70). Insulin may act indirectly to stop WMH progression by preventing damage caused by hyperphosphorylated tau, Aβ40, and Aβ42 to both the blood brain barrier and myelin producing oligodendrocytes. Further research needs to be done to elucidate patterns behind regional WMH load and CSF AD biomarkers of Aβ and tau.

Numerous studies have sought to identify the temporal and spatial patterns of gray matter volume loss in typical AD progression (71). While there is not a consensus across all studies, several vulnerable regions have been highlighted (11, 72). Measures of gray matter volume and thickness were unchanged by 12 months of INI administration. It is possible that our region of interest, while widely accepted, failed to capture subtle differences between treatment groups. Other regions of the brain that were excluded from our analysis may be more sensitive to changes specific to this intervention. Previous interventions have shown decreased rates of atrophy in patients with MCI treated with other noninsulin interventions; however, these findings were over a 2-year period (73). It is possible that our intervention was not long enough to detect subtle changes in gray matter volume. Cognitive improvement has been observed without halting gray matter atrophy. A phase II clinical trial testing daily administration of resveratrol showed greater reduction in brain volume in the treatment group compared to placebo after one year of treatment, but the resveratrol-treated group also showed less decline on the ADCS-ADL suggesting interventions can still be beneficial independent of apparent reduction of gray matter volume (74).

Our study had several limitations. Our small sample size may have contributed to our inability to detect preservation of gray matter volume and cortical thickness by INI treatment, and may also have reduced our ability to detect relationships between WMHVs and other measures. Our cohort was predominantly white (93%) and male (62%) and thus results may not generalize to a more diverse population. A number of participants did not complete the trial or had usable data at either baseline or follow up. These missing datapoints could result in a completer bias; however, this is unlikely as those with incomplete data did not differ demographically or in any baseline measures from completers. It is also possible that a longer intervention may be needed to observe a divergence between groups on our measures. These results need to be replicated in a larger, longer study in order to determine the effects of insulin on the brain WM health and the mechanistic pathways underlying these effects.

In conclusion, we found that treating MCI and AD patients with 12 months of INI significantly reduced WMH progression without affecting gray matter volume or cortical thickness, and that increases in WMHV correlated with both worsening in AD CSF biomarker profile and cognitive/functional measures. These findings support insulin's potential as a therapeutic option for AD; however, more research needs to be conducted to elucidate mechanism through which insulin may impact white matter integrity.

## Electronic supplementary material

Supplemental Table 1. Raw means and confidence intervals for temporal-parietal ROI volume and thickness change split by treatment group.

Supplemental Table 2. Raw means for global and regional white matter hyperintensity volumes at screening and month 12 follow-up for insulin and placebo groups.

*Ethical Standards:* The study was approved by the Institutional Review Boards of all participating institutions. All participants provided written informed consent to participate in the study.
