# Supplementary material for: Intranasal Insulin Reduces White Matter Hyperintensity Progression in Association with Improvements in Cognition and CSF Biomarker Profiles in Mild Cognitive Impairment and Alzheimer's Disease
Source: J Prev Alzheimers Dis. 2021 Apr 7;8(3):240–8. doi: 10.14283/jpad.2021.14 (PMC10233712; doi:10.14283/jpad.2021.14)
Supplement: Supplementary file 1 — Supplemental Table 1. Raw means and confidence intervals for temporal-parietal ROI volume and thickness change split by treatment group. [file mmc1.docx]

Supplemental Table 1. Raw means and confidence intervals for temporal-parietal ROI volume and thickness change split by treatment group.

| **Region** | **Arm** | **Mean Baseline** | **Mean Month 12** |
| --- | --- | --- | --- |
| Temporal-Parietal ROI Volume, cm^3^ | Insulin | 93.22 | 88.71 |
|  | Placebo | 91.17 | 86.63 |
| Temporal-Parietal ROI Thickness, mm | Insulin | 2.61 | 2.53 |
|  | Placebo | 2.52 | 2.43 |
