# Supplementary material for: Intranasal Insulin Reduces White Matter Hyperintensity Progression in Association with Improvements in Cognition and CSF Biomarker Profiles in Mild Cognitive Impairment and Alzheimer's Disease
Source: J Prev Alzheimers Dis. 2021 Apr 7;8(3):240–8. doi: 10.14283/jpad.2021.14 (PMC10233712; doi:10.14283/jpad.2021.14)
Supplement: Supplementary file 2 — Supplemental Table 2. Raw means for global and regional white matter hyperintensity volumes at screening and month 12 follow-up for insulin and placebo groups. [file mmc2.docx]

Supplemental Table 2. Raw means for global and regional white matter hyperintensity volumes at screening and month 12 follow-up for insulin and placebo groups.

| **Region** | **Arm** | **Mean Baseline WMHV, cm^3^** | **Mean Month 12 WMHV, cm^3^** |
| --- | --- | --- | --- |
| Global | Insulin | 26.31 | 30.30 |
|  | Placebo | 13.19 | 17.24 |
| Deep WM | Insulin | 1.04 | 1.21 |
|  | Placebo | 0.58 | 0.92 |
| SSCorpus Callosum | Insulin | 0.97 | 1.12 |
|  | Placebo | 0.84 | 1.03 |
| Temporal Lobe | Insulin | 1.69 | 1.93 |
|  | Placebo | 0.92 | 1.18 |
| Occipital Lobe | Insulin | 3.06 | 3.34 |
|  | Placebo | 1.62 | 1.91 |
| Parietal Lobe | Insulin | 7.08 | 8.48 |
|  | Placebo | 4.19 | 5.39 |
| Frontal Lobe | Insulin | 12.47 | 14.22 |
|  | Placebo | 5.03 | 6.82 |
