# Supplementary material for: Intranasal Insulin Reduces White Matter Hyperintensity Progression in Association with Improvements in Cognition and CSF Biomarker Profiles in Mild Cognitive Impairment and Alzheimer’s Disease
Source: J Prev Alzheimers Dis. Author manuscript; Available in PMC 2023 Jun 1. (PMC10233712; doi:10.14283/jpad.2021.14)
Supplement: Supplementary file 1 [file NIHMS1892908-supplement-supp_1.pdf]

Supplemental Table 1. Raw means and confidence intervals for temporal-parietal ROI volume and thickness change split by treatment group.

| Region | Arm | Mean Baseline | Mean Month 12 |
|---|---|---|---|
| Temporal-Parietal ROI Volume, $cm^3$ | Insulin | 93.22 | 88.71 |
| | Placebo | 91.17 | 86.63 |
| Temporal-Parietal ROI Thickness, mm | Insulin | 2.61 | 2.53 |
| | Placebo | 2.52 | 2.43 |
