# Supplementary material for: Intranasal Insulin Reduces White Matter Hyperintensity Progression in Association with Improvements in Cognition and CSF Biomarker Profiles in Mild Cognitive Impairment and Alzheimer’s Disease
Source: J Prev Alzheimers Dis. Author manuscript; Available in PMC 2023 Jun 1. (PMC10233712; doi:10.14283/jpad.2021.14)
Supplement: Supplementary file 2 [file NIHMS1892908-supplement-supp_2.pdf]

Supplemental Table 2. Raw means for global and regional white matter hyperintensity volumes at screening and month 12 follow-up for insulin and placebo groups.

Region	Arm	Mean Baseline WMHV, cm^3	Mean Month 12 WMHV, cm^3
Global	Insulin	26.31	30.30
	Placebo	13.19	17.24
Deep WM	Insulin	1.04	1.21
	Placebo	0.58	0.92
SSCorpus Callosum	Insulin	0.97	1.12
	Placebo	0.84	1.03
Temporal Lobe	Insulin	1.69	1.93
	Placebo	0.92	1.18
Occipital Lobe	Insulin	3.06	3.34
	Placebo	1.62	1.91
Parietal Lobe	Insulin	7.08	8.48
	Placebo	4.19	5.39
Frontal Lobe	Insulin	12.47	14.22
	Placebo	5.03	6.82
